# Factors Increasing the Likelihood of Postoperative Hematomas Following Thyroid Surgery

**DOI:** 10.1002/hed.28096

**Published:** 2025-02-12

**Authors:** Emily Ajit‐Roger, Jessica Hier, Marco Mascarella, Koorosh Semsar‐Kazerooni, Sabrina Daniela Silva Wurzba, Véronique‐Isabelle Forest, Michael P. Hier, Alex Mlynarek, Carmelina Mancini, Richard J. Payne

**Affiliations:** ^1^ Faculty of Medicine and Health Sciences McGill University Montréal Quebec Canada; ^2^ Department of Otolaryngology – Head and Neck Surgery, Faculty of Medicine McGill University Montréal Quebec Canada; ^3^ Department of Otolaryngology – Head and Neck Surgery, Jewish General Hospital McGill University Montréal Quebec Canada; ^4^ Department of Otolaryngology – Head and Neck Surgery, Royal Victoria Hospital McGill University Montréal Quebec Canada; ^5^ Centre for Clinical Epidemiology Lady Davis Institute of the Jewish General Hospital Montréal Quebec Canada; ^6^ Segal Cancer Centre and Lady Davis Institute for Medical Research, Department of Medicine and Department of Oncology, Sir Mortimer B. Davis‐Jewish General Hospital, Faculty of Medicine McGill University Montréal Quebec Canada

**Keywords:** neck hematoma, outpatient surgery, retrospective review, surgical complications, thyroid surgery

## Abstract

**Background:**

Neck hematoma following thyroid surgery is a potentially life‐threatening complication.

**Methods:**

This retrospective case–control study reviewed neck hematoma reoperations following thyroid surgery (2009–2024), using 3:1 matching. Univariable analysis identified hematoma and delayed onset (≥ 6 h) risk factors, with odds ratios (ORs) and 95% confidence intervals (CIs).

**Results:**

Among 5502 surgeries, the hematoma incidence was 0.55% (*n* = 30). The mean age was 54 and the female‐to‐male ratio was 7:3. Key risk factors included pre‐induction blood pressure > 160 mmHg (OR = 3.04 [95% CI = 1.25–7.39], *p* = 0.014) and limited blood pressure change postmedication (OR = 6.25 [95% CI = 1.03–38.08], *p* = 0.047). The hematoma group had higher rates of smoking, hypertension, diabetes, Graves' disease, and prior thyroid surgery, and, in delayed hematoma cases, larger nodules, total thyroidectomy, and central neck dissection, though not statistically significant.

**Conclusion:**

Patients with poorly controlled blood pressure may not be candidates for outpatient thyroidectomy.

## 1 Background

Thyroidectomy is a commonly performed surgical procedure done for both benign and malignant conditions, including thyroid nodules, Graves' disease, and thyroid cancer [1]. Surrounding structures, such as the recurrent laryngeal nerves and parathyroid glands, are at risk of injury during this surgical procedure [1]. The incidence of postoperative hematoma formation, a rare but potentially life‐threatening complication, ranges between 0.7% and 5% [2].

With resource constraints in the public health care system [3, 4], investigating the safety of performing outpatient thyroidectomies as a strategic measure to alleviate the system's burden is crucial. Among the spectrum of complications that may occur following thyroid surgery, hematomas are of paramount concern due to their impact on the airway, which can prove fatal. Hence, the examination of risk factors for the development of this complication is also critical. While there exists a vast body of literature addressing risk factors associated with hematoma development, there is a notable scarcity in scrutinizing this topic from a public healthcare perspective. Current literature suggests that male sex, older age, hypertension, Graves' disease, antiplatelet and anticoagulant use, prior thyroid surgery, total thyroidectomy, low volume centers, and any concomitant lymph node neck dissection [5] have been identified as contributing to the heightened risk of hematoma formation following thyroid surgery.

This nested case–control retrospective study at a single high‐volume thyroid surgery institution examines the cases of patients who have suffered from a neck hematoma following thyroid surgery, requiring surgical intervention. The primary objective of this study is to identify potential perioperative risk factors for postoperative neck hematomas. The secondary objective of this study is to compare characteristics surrounding early vs. late hematoma development. This study intends to contribute to the existing body of literature aimed at differentiating between the cohort of patients who are at higher risk of postsurgical hematoma development and those who would be suitable for undergoing thyroid surgery in an outpatient setting.

## 2 Methods

### 2.1 Study Design

This study is a single‐institution retrospective nested case–control study following the STROBE guidelines [6]. It identified all patients who suffered a hematoma after thyroid surgery requiring a return to the operating room, in the last 15 years. The study took place at the Jewish General Hospital in Montreal, Canada.

Ethics approval was obtained by the Medical‐Bioethics Research Ethics Committee (REC) of the Integrated Health and Social Services University Network for West‐Central Montreal (#2023‐3679). Cases were identified using data from five otolaryngologists, mainly using the National Surgical Quality Improvement Program (NSQIP) database, personal surgeon logs, and the institution's operating room records. At our institution, standard clips are exclusively used for hemostasis, while vessel ligatures and harmonic scalpels are not used. The clips used are the Weck Horizon Ligating Clips.

### 2.2 Patient Population

The charts of adult patients (18 years and older) who suffered a hematoma after thyroid surgery were reviewed from February 2009 to February 2024. Patients who developed a clinically diagnosed neck hematoma which required a return to the operating room were included. Patients with lateral neck dissections were excluded, as this subset of patients would not have been considered for outpatient surgery due to the expected extent of surgery. Thirty patients in total were included.

### 2.3 Control Population

The charts of adult patients who underwent thyroid surgery without developing a hematoma were reviewed from February 2009 to February 2024. For each case with postoperative bleeding, three control cases were matched based on the surgeon, year of surgery (±2 years), thyroid pathology, sex, and age (±10 years or within the following age ranges < 30, 30–50, 50–70, or > 70). The same exclusion criteria utilized for the patient population were applied to the control group. The matching parameters were carefully selected based on evidence from the literature of known confounders and preserving the ability to analyze other potential risk factors.

### 2.4 Data Collection

Data were collected using operating room dictation reports, pathology reports, anesthesia records, postanesthesia care unit progress notes, and inpatient surgical ward progress notes. The outcome measure was defined as a clinically diagnosed neck hematoma that required a return to the operating room. The exposures evaluated included comorbidities, thyroid pathology, and perioperative hemodynamics.

Collected data from the electronic medical records included patient demographics (age, sex, body mass index, American Society of Anesthesiologist [ASA] score, active smoking history, history of hypertension, diabetes mellitus, bleeding disorder, previous neck radiation, previous thyroid surgery or use of anticoagulants within 7 days preoperatively), thyroid pathology, surgical factors (surgeon, anesthesiologist, duration of surgery, and surgical extent), and perioperative circumstances (timing of hematoma development, perioperative hemodynamics, inciting factors, and length of hospitalization). The time interval for occurrence of hematoma was measured as the time interval between the time the patient left the operating room to the first documentation of neck swelling. Additionally, the total number of thyroid surgeries performed per surgeon since 2009 at this institution was retrieved.

### 2.5 Statistical Analysis

Statistical analysis was performed using IBM SPSS Statistics Version 29.0.1.1 (SPSS Inc., Chicago, IL, USA) software. Statistical significance was defined as *p* value ≤ 0.05.

Descriptive statistics, including mean, standard deviation, and frequency distributions, were performed for all collected variables. Continuous data were presented as mean ± standard deviation (SD) and tested by one‐way ANOVA. Categorical data were presented as *N* (%) and tested using a chi‐square test.

Risk factors for postoperative bleeding were evaluated using univariable conditional logistic regression and are presented as odds ratios (ORs) with 95% confidence intervals (CIs). Receiver operating characteristic (ROC) curves were generated to assess the predictive performance of postoperative blood pressure and heart rate for the development of hematoma. Using the area under the curve (AUC), the Youden's index was utilized to determine the optimal cutoff values for postoperative blood pressure and heart rate.

Risk factors for late hematoma development were evaluated using univariable conditional logistic regression and are presented as ORs with 95% CIs. Univariable analysis was conducted to discern the significance of factors concerning the timing of postoperative hematomas (< 6 h or ≥ 6 h).

## 3 Results

### 3.1 Patient Demographics

Out of 5502 thyroid surgeries performed over the past 15 years, 30 patients had a hematoma in this study, resulting in an incidence of 0.55%. Within the hematoma group, there was a higher rate of smoking, hypertension, type II diabetes, and prior thyroid surgery, as depicted in Table 1.

**TABLE 1:** Univariable analysis of demographic risk factors associated with hematoma.

Demographic characteristics	Postoperative hematoma, *n* = 30 (%)	Control group, *n* = 90 (%)	Univariable analysis, OR (95% CI)	*p*
Age (mean, SD)	53.6 ± 17.7	55.5 ± 14.7	0.99 (0.97–1.02)	0.574
Sex				
Female	21 (70%)	63 (70%)	1 (0.41–2.46)	1.000
Male	9 (30%)	27 (30%)		
BMI^a^ (mean, SD)	26.9 ± 5.2	27.4 ± 4.9	0.98 (0.89–1.07)	0.606
Active smoker	5 (16.7%)	6 (6.7%)	2.8 (0.79–9.95)	0.112
Comorbidities				
Hypertension	13 (43.3%)	29 (32.2%)	1.61 (0.69–3.75)	0.271
Diabetes type II	4 (13.3%)	5 (5.6%)	2.61 (0.65–10.46)	0.174
Bleeding disorders	0	0	N/A	N/A
Prior thyroid surgery	5 (16.7%)	7 (7.8%)	2.37 (0.69–8.12)	0.169
ASA score				
ASA I	7 (23.3%)	20 (22.2%)		
ASA II	18 (60%)	60 (66.7%)	0.86 (0.31–2.35)	0.765
ASA III	5 (16.7%)	10 (11.1%)	1.43 (0.36–5.66)	0.611
Previous neck radiation	0	0	N/A	N/A
Anticoagulants^b^	0	1 (1.11%)	N/A	N/A

^a^ Body mass index = kg/m^2^.

^b^ Oral anticoagulant or antiplatelet use within 7 days of operation.

### 3.2 Surgical Factors

Missing data for pre‐induction and intraoperative hemodynamics were noted for one patient in the hematoma group.

A pre‐induction systolic blood pressure exceeding 160 mmHg was significantly associated with an increased risk of hematoma, with an OR of 3.04 (95% CI = 1.25–7.39). In the hematoma group, there was a higher rate of intraoperative systolic blood pressure exceeding 160 mmHg, thyroid hyperplasia, large nodules, and subtotal/completion thyroidectomy, as depicted in Table 2. Notably, 13.3% of patients in the hematoma group had Graves' disease, compared to an overall rate of 3.6% among all patients who underwent thyroid surgery over the last 15 years. Details regarding the surgeon and anesthesiologist involved can be found in Supporting Information: Supplement 1 and Supplement 2, respectively.

**TABLE 2:** Univariable analysis of operative risk factors associated with hematoma.

Operative characteristics	Postoperative hematoma, *n* = 30 (%)	Control group, *n* = 90 (%)	Univariable analysis, OR (95% CI)	*p*
Surgical duration^a^ (mean, SD)	70.7 ± 30.7	72 ± 27.8	1.00 (0.98–1.01)	0.830
Late surgical start (≥ 14 h)	2 (6.7%)	15 (16.7%)	0.26 (0.07–1.66)	0.189
BP^b^ > 160 mmHg				
Pre‐induction	13 (44.8%)	19 (21.1%)	3.04 (1.25–7.39)	0.014
Intraoperative	9 (31%)	22 (24.4%)	1.39 (0.55–3.50)	0.483
Comorbid hypertension and				
Preoperative BP > 160 mmHg	7 (53.8%)	9 (31%)	2.59 (0.68–9.95)	0.165
Intraoperative BP > 160 mmHg	5 (38.5%)	12 (41.4%)	0.89 (0.23–3.38)	0.859
Pathology findings^c^				
Papillary thyroid cancer	21 (70%)	72 (80%)	0.58 (0.23–1.49)	0.259
Adenoma	7 (23.3%)	25 (27.8%)	0.79 (0.30–2.07)	0.634
Hyperplasia	5 (16.7%)	8 (8.9%)	2.05 (0.62–6.83)	0.242
Graves' disease	4 (13.3%)	12 (13.3%)	1.00 (0.30–3.37)	1.000
CLT^d^	8 (26.7%)	32 (35.6%)	0.66 (0.26–1.65)	0.373
TNM^e^ tumor stage				
T1	7 (50%)	32 (59.3%)	N/A	N/A
T2	6 (42.9%)	14 (25.9%)	1.96 (0.56–6.90)	0.295
T3	1 (7.1%)	8 (14.8%)	0.57 (0.06–5.34)	0.623
Dominant nodule size^f^ (mean, SD)	2.6 ± 1.8	2.4 ± 1.4	1.12 (0.83–1.52)	0.447
Surgical extent				
Hemi thyroidectomy	14 (46.7%)	30 (33.3%)	1.18 (0.76–4.06)	0.192
Total thyroidectomy	12 (40%)	55 (61.1%)	0.42 (0.18–0.99)	0.047
Completion thyroidectomy	4 (13.3%)	5 (5.6%)	2.62 (0.65–10.46)	0.174
Central neck dissection level VI	16 (53.3%)	58 (64.4%)	0.63 (0.27–1.46)	0.280
Parathyroidectomy	8 (26.7%)	41 (45.6%)	0.44 (0.18–1.08)	0.072
Presence of neck drain	1 (3.3%)	3 (3.3%)	1.00 (0.1–10)	1.000
Blood loss^g^	0	0	N/A	N/A

^a^ Surgical duration in minutes.

^b^ Systolic blood pressure.

^c^ Intraoperative surgical pathology, including all pathology findings.

^d^ Chronic lymphocytic thyroiditis.

^e^ Extent of the size of the tumor (T) per the American Joint Committee on Cancer staging system.

^f^ Dominant nodule size regardless of malignancy status in centimeters.

^g^ Blood loss reported as minimal or less than 30 mL was reported as 0.

### 3.3 Perioperative Hemodynamics

Among the hematoma group, one patient developed the hematoma immediately after surgical closure in the operating room, resulting in no available postoperative hemodynamic data for this case.

A postoperative maximal heart rate below 90 beats per min was significantly associated with an increased risk of hematoma, with an OR of 3.14 (95% CI = 1.22–8.09). When accompanied by comorbid hypertension, the risk of hematoma increased with an OR of 26.67 (95% CI = 3.0–237.42). A nonsignificant change in blood pressure following medication administration was significantly associated with an increased risk of hematoma, with an OR of 6.25 (95% CI = 1.03–38.08). Within the hematoma group, there was a higher rate of postoperative blood pressure below 120 mmHg, postoperative blood pressure exceeding 160 mmHg, and both intraoperative and postoperative episodes requiring antihypertensive medications, as depicted in Table 3.

**TABLE 3:** Univariable analysis of postoperative risk factors associated with hematoma.

Postoperative characteristics	Postoperative hematoma, *n* = 30 (%)	Control group, *n* = 90 (%)	Univariable analysis, OR (95% CI)	*p*
Postoperative BP^a^				
≤ 120 mmHg	2 (6.9%)	4 (4.4%)	1.59 (0.28–9.18)	0.603
121–140 mmHg	8 (27.6%)	27 (30%)	0.89 (0.35–2.26)	0.804
141–160 mmHg	9 (31%)	36 (40%)	0.68 (0.28–1.65)	0.388
> 160 mmHg	10 (34.5%)	23 (25.6%)	1.53 (0.62–3.78)	0.352
Postoperative HR^b^				
≤ 90 bpm	22 (75.9%)	44 (66.7%)	3.14 (1.22–8.09)	0.014
> 90 bpm	7 (13.2%)	46 (51.1%)		
Comorbid hypertension and				
Postoperative BP > 160 mmHg	6 (38.1%)	10 (34.5%)	1.63 (0.43–6.17)	0.473
Postoperative HR ≤ 90 bpm	12 (92.3%)	9 (31%)	26.67 (3.0–237.42)	0.003
Intraoperative vasopressors				
Patients	4 (13.3%)	15 (16.7%)	1.0 (0.33–30.03)	1.000
Number of episodes^c^ (mean, SD)	1 ± 0	1.7 ± 0.6	0.61 (0.26–1.47)	0.271
Premedication HR (mean, SD)	69 ± 10.8	61.2 ± 9.7	1.1 (0.98–1.19)	0.144
Premedication BP (mean, SD)	85 ± 5.8	83.6 ± 12.6	1.0 (0.92–1.10)	0.894
Postmedication^d^ HR (mean, SD)	63.8 ± 4.8	67.8 ± 15.9	0.98 (0.89–1.07)	0.609
Postmedication BP (mean, SD)	107.5 ± 9.6	115.2 ± 19.6	0.97 (0.91–1.04)	0.394
Intraoperative antihypertensives				
Patients	3 (1%)	3 (3.3%)	1.0 (0.10–10.0)	1.000
Number of episodes (mean, SD)	1.7 ± 1.5	1.3 ± 0.6	2.12 (0.74–6.09)	0.163
Premedication HR (mean, SD)	83 ± 22.9	85 ± 17.9	0.98 (0.92–1.04)	0.518
Premedication BP (mean, SD)	163 ± 17.2	152 ± 17.9	1.05 (0.96–1.15)	0.328
Postmedication HR (mean, SD)	70 ± 11.7	74 ± 11.4	0.933 (0.83–1.05)	0.245
Postmedication BP (mean, SD)	122 ± 19.2	117 ± 15.7	1.02 (0.94–1.11)	0.622
Postoperative PRN antihypertensives^e^				
Patients	5 (16.7%)	13 (14.44%)	1.19 (0.38–3.65)	0.768
Number of episodes (mean, SD)	2.4 ± 1.1	1.5 ± 0.7	1.36 (0.81–2.29)	0.250
Labetalol doses per episode^f^ (mean, SD)	2.8 ± 1.8	2.1 ± 0.6	1.32 (0.86–2.03)	0.213
Premedication HR (mean, SD)	80 ± 10.7	75.1 ± 15.8	1.00 (0.94–1.06)	0.916
Postmedication HR (mean, SD)	77.6 ± 13.2	76.3 ± 20.6	0.99 (0.95–1.04)	0.690
Change in HR (mean, SD)	−1.7 ± 16.9	−2.7 ± 20	1.00 (0.95–1.05)	0.837
Nonsignificant change^g^ (> 6.5)	N/A	N/A	1.3 (0.23–7.32)	0.766
Premedication BP (mean, SD)	151.8 ± 6.7	171 ± 15.1	0.83 (0.72–0.96)	0.010
Postmedication BP (mean, SD)	147 ± 14.4	154.2 ± 16	0.95 (0.90–1.02)	0.150
Change in BP (mean, SD)	−7.6 ± 12	−17.7 ± 12	1.05 (0.94–1.18)	0.410
Nonsignificant change (> −8)	N/A	N/A	6.25 (1.03–38.08)	0.047
Postoperative usual antihypertensives^h^				
Patients	1 (3.3%)	9 (1%)	0.31 (0.04–2.56)	0.277
Number of drugs (mean, SD)	1 ± 0	1.2 ± 0.4	N/A	N/A
Premedication HR (mean, SD)	80 ± 0	93.4 ± 22.7	0.96 (0.82–1.11)	0.548
Premedication BP (mean, SD)	160 ± 0	143.6 ± 23.8	1.05 (0.89–1.25)	0.532
Postmedication HR (mean, SD)	N/A	86.4 ± 18.1	N/A	N/A
Postmedication BP (mean, SD)	N/A	123.4 ± 16.5	N/A	N/A

^a^ As defined as the maximal systolic blood pressure measurement before hematoma documentation or within 6 h postoperatively.

^b^ As defined as the maximal heart rate measurement before hematoma documentation or within 6 h postoperatively.

^c^ Defined as episodes of hemodynamic instability requiring medication administration separated by at least 30 min.

^d^ Measured at the peak action time of the medication.

^e^ Defined as the administration of a rapid‐acting antihypertensive medication on an as‐needed basis on the same day postoperatively or before hematoma development.

^f^ Defined as the number of repeated doses of Labetalol required per episode.

^g^ Significant change was determined based on Youden's index, with values above the 75th percentile, indicating nonsignificant postmedication changes.

^h^ Defined as the administration of a patient's prescribed at‐home antihypertensive medications on the same day postoperatively or before hematoma development.

### 3.4 Context of Hematoma Development

**FIGURE 1:**
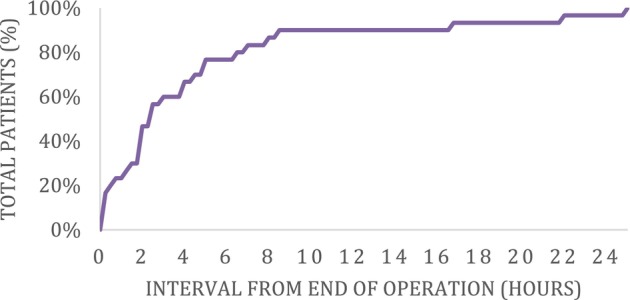
Cumulative curve for time to hematoma development. [Color figure can be viewed at wileyonlinelibrary.com]

The average duration from surgical closure to the clinical detection of hematoma was 209 min (SD 252.306), ranging from 5 min to 25 h (Figure 1). Initial documentation of neck hematoma occurred in the postanesthesia care unit in 60% (18 out of 30) of patients, in the postoperative surgical unit in 36.7% (11 out of 30) of patients, and one patient developed the hematoma in the operating room, immediately after surgical closure. None of the patients had documented events of increased intrathoracic pressure, such as coughing, sneezing, vomiting, rapid neck movements, or excessive mobilization before their hematoma.

### 3.5 Late Hematoma Development

In the delayed hematoma presentation group, there was a higher rate of large nodules, thyroid hyperplasia, total thyroidectomy, central neck dissection, parathyroidectomy, intraoperative blood pressure over 160 mmHg and postoperative heart rate below 90 bpm (Table 4).

**TABLE 4:** Univariable analysis of risk factors associated with late hematoma.

Variables	Less than 6 h, *n* = 25 (%)	6 h or greater, *n* = 5 (%)	Univariable analysis, OR (95% CI)	*p*
Patient demographics				
Age (mean, SD)	53.6 ± 18.4	53.6 ± 15.3	1.00 (0.95–1.06)	0.996
BMI (mean, SD)	26.8 ± 4.8	27.2 ± 7.8	1.02 (0.84–1.22)	0.870
Male sex	8 (32%)	1 (20%)	0.53 (0.05–5.53)	0.597
Smoker	4 (16%)	1 (20%)	0.76 (0.07–8.73)	0.827
Hypertension	11 (45%)	2 (40%)	0.85 (0.12–6.00)	0.869
Diabetes type II	3 (12%)	1 (20%)	1.8 (0.15–22.37)	0.635
Previous thyroid surgery	4 (16%)	1 (20%)	1.31 (0.12–15.03)	0.827
Dominant nodule size^a^ (mean, SD)	2.5 ± 2	2.8 ± 1.3	1.09 (0.63–1.87)	0.770
Thyroid pathology				
Papillary thyroid cancer	16 (64%)	4 (80%)	1.88 (0.18–19.68)	0.597
Unspecified adenomas	6 (24%)	1 (20%)	0.79 (0.07–8.52)	0.847
Hyperplasia	3 (12%)	2 (40%)	4.89 (0.57–42.3)	0.149
Graves' disease	4 (16%)	0	N/A	N/A
Chronic lymphocytic thyroiditis	5 (20%)	2 (40%)	2.11 (0.28–15.77)	0.466
Surgical timing > 14 h	2 (8%)	0	N/A	N/A
Surgical extent				
Hemi	13 (52%)	1 (20%)	0.23 (0.02–2.37)	0.217
Total	9 (36%)	3 (60%)	2.67 (0.37–19.06)	0.328
Completion	3 (12%)	1 (20%)	1.83 (0.15–22.37)	0.635
Central neck dissection Level VI	12 (48%)	4 (80%)	4.33 (0.42–44.43)	0.217
Parathyroidectomy	6 (24%)	2 (40%)	2.11 (0.28–15.77)	0.466
Duration of surgery^b^ (mean, SD)	72.9 ± 33.3	60 ± 7.1	0.98 (0.93–1.03)	0.400
Perioperative hemodynamics				
Preoperative BP > 160 mmHg	11 (44%)	2 (40%)	0.79 (0.11–5.60)	0.812
Intraoperative BP > 160 mmHg	7 (28%)	2 (40%)	1.62 (0.22–11.89)	0.636
Postoperative BP > 160 mmHg	9 (36%)	1 (20%)	0.42 (0.04–4.33)	0.464
Postoperative HR ≤ 90 bpm	17 (56.7%)	5 (100%)	N/A	N/A
Intraoperative antihypertensives				
Patients	3 (1%)	0	N/A	N/A
Number of episodes (mean, SD)	1.7 ± 1.5	N/A	N/A	N/A
Postoperative PRN antihypertensives				
Patients	4 (16%)	1 (20%)	N/A	N/A
Number of episodes (mean, SD)	2 ± 0.8	4 ± 0	N/A	N/A
Length of hospital stay^c^ (mean, SD)	57.6 ± 35.2	135.6 ± 154.1	1.01 (1.00–1.03)	0.158

^a^ Dominant nodule size regardless of malignancy status in centimeters.

^b^ Surgical duration in minutes.

^c^ Length of hospitalization from end of surgery till discharge in hours.

## 4 Discussion

This comprehensive investigation into the demographic and perioperative factors associated with cervical hematomas following thyroid surgery provides valuable insights. This study benefited from valuable input from surgeons, anesthesiologists, and PACU room nurses, whose perspectives helped evaluate the risk of hematoma from multiple lenses. By matching cases based on factors such as surgical technique, age, sex, and thyroid pathology, our study was able to control for potential confounders, allowing a more nuanced exploration of the underlying causes of hematoma development. The matching criteria were intentionally selected to control for key nonmodifiable confounders while preserving the ability to analyze modifiable risk factors. Male sex, older age, and Graves' disease are well‐established risk factors for neck hematoma after thyroid surgery [7]. Since these factors are relatively underrepresented in the general thyroid surgery population, matching for sex, age, and thyroid pathology ensured that sufficient inclusion of high‐risk patients in the control group allowed for meaningful comparisons and adequate control for these nonmodifiable confounders. Additionally, given that our study spans 15 years, surgical techniques and perioperative care protocols have evolved during this time frame and, matching by year of surgery minimized potential temporal biases. Finally, surgeon‐specific variations in technique can influence outcomes and are challenging to quantify, and therefore matching by surgeons helps to mitigate this variability. While surgical extent is a recognized potential cofounder [7], overmatching could obscure important independent associations between surgical extent and hematoma risk, one of the factors aimed to be investigated in our study. Overall, our approach was designed to identify modifiable risk factors, providing actionable insights to reduce hematoma risk in clinical practice.

Comorbidities such as hypertension and type II diabetes are well‐established risk factors for hematoma development. In accordance with the findings of Fan et al. [7] and Mahoney et al. [8], our study revealed higher rates of both conditions in the hematoma group. Additionally, we observed a potential connection between higher ASA scores and patients who developed hematomas. This aligns with a large multicenter study by Foley et al. [9], which identified ASA scores as independent predictors of postoperative complications in outpatient surgeries. These results underscore the importance of considering ASA scores when determining whether a patient is suitable for outpatient thyroid surgery, as these scores incorporate medical comorbidities and are associated with postoperative complications.

When considering any surgical complication, one must consider the experience of the surgeon. Dehal et al.'s [10] 10‐year analysis demonstrated that high‐volume surgeons, those who performed over 100 thyroid surgeries, had lower complication rates. However, since all surgeons in our study would be classified as high‐volume surgeons, this precludes the ability to assess any linear relationship between surgical volume and hematoma rates. The overall low hematoma rate observed in our study compared to other rates in the literature [2] may be attributed to the expertise of the participating surgeons, further emphasizing the importance of surgical experience in minimizing complications.

Characteristics of thyroid pathology and their correlation with surgical complications have been previously explored in the literature. A meta‐analysis by Quimby et al. [11] identified a significant increase in hematoma risk among patients with Graves' disease, a finding supported by our study, where a high prevalence of Graves' disease was observed in the hematoma group. Additionally, while the association between malignant thyroid disease and hematoma risk remains contentious [7], the true risk appears to lie in the size of the tumor, rather than the malignancy status [12, 13]. In our study, patients in the hematoma group had a greater dominant nodule size, supporting the notion that patients with thyroid conditions necessitating more extensive surgical interventions may likely not be ideal candidates for outpatient surgery.

This consideration prompts an exploration of the influence of surgical extent on the occurrence of neck. A 2019 meta‐analysis by Fan et al. identified that total thyroidectomy and any level of neck dissection [7] increase the risk for hematoma development. Conversely, our study found an inverse relationship between total thyroidectomy and hematoma development, likely attributed to lower rates of comorbidities in patients who underwent a total thyroidectomy.

Several patient factors must be considered in the perioperative period, particularly altered hemodynamics. A retrospective case–control analysis by Morton and Vandal, published nearly a decade ago, identified that for every 10‐point rise in systolic blood pressure, there was a 39% increase in the risk of bleeding [14], underscoring the now well‐established importance of vigilant postoperative blood pressure management. Additionally, a comprehensive literature review published in the American Heart Association journal [15] emphasizes the significance of blood pressure management during the intraoperative phase, highlighting that systolic blood pressure measurements exceeding 160 mmHg are independently associated with extended hospital stays and increased mortality [15]. Accordingly, our study adopted 160 mmHg as a crucial cutoff measure of blood pressure. Notably, a significant portion of patients in our study who developed a hematoma exceeded these recommended blood pressure thresholds during intraoperative and postoperative periods. Unexpectedly, we observed a markedly significant association between elevated pre‐induction blood pressure and hematoma development, an area for which less is known. This finding suggests that diligent blood pressure control should be implemented not only in the postoperative period but also in the period preceding surgery.

Building on our perioperative hemodynamic findings, we further investigated the role of medication administration. Interestingly, we observed that patients who did not exhibit significant blood pressure reductions after receiving postoperative antihypertensives were at higher risk of developing hematomas. Additionally, postoperative heart rate below 90, particularly in patients with comorbid hypertension, was associated with an increased hematoma risk. Hypertension is known to cause vascular dysfunction, characterized by increased vascular contraction and arterial remodeling, led by pro‐hypertensive stimuli such as activation of the sympathetic nervous activation and hemodynamic shifts [16]. A reduced heart rate may indicate an impaired autonomic response, and when combined with underlying vascular dysfunction, it could compromise one's ability to adequately perform hemostasis.

Further, it is sensible to investigate potential precipitating factors. A retrospective study by Chen et al. revealed that upper pole bleeding sources were often associated with sudden violent coughing, sneezing, or vomiting, especially during extubation [17]. While our retrospective study sought to identify such inciting events, no documented occurrence of events causing increased intrathoracic pressure was noted for any of the patients. This likely reflects insufficient documentation, a key limitation of our study. Despite this, this finding does not preclude the existence of a potential trigger, warranting further investigation in future studies.

Appreciating the typical timeline for hematoma development must be understood to safely plan the duration of postoperative monitoring. In a multi‐institutional case–control study by Campbell et al., the mean time to hematoma formation was 7 h, spanning from 0 min to 9 days, with nearly half of the patients returning to the operating room within 6 h [18]. Accordingly, our study stratified the timing of hematoma onset using a 6‐h mark. Notably, our findings revealed a notably shorter period, with half of the cases occurring within 2 h postoperatively. Additionally, there are higher rates of delayed hematoma development in patients with a total thyroidectomy, neck dissection, or large thyroid nodules. This may suggest that a delayed presentation is associated with larger surgical cavities, masking earlier signs of bleeding.

Outpatient total and subtotal thyroidectomies are becoming increasingly popular and are associated with low complication rates [19, 20]. The decision to perform thyroid surgery in an outpatient setting necessitates careful evaluation. Factors such as the expertise and experience of the surgical team, the patient's health profile, and the features of thyroid pathology, along with its anticipated surgical plan [21] need to be prudently considered. The input from surgeons, anesthesiologists, and PACU nurses was essential to comprehensively evaluate this rare but critical complication from multiple perspectives, highlighting the importance of a multidisciplinary approach in the perioperative setting. The patient's social circumstances, including having a responsible adult accompany them overnight, living within reasonable proximity to a suitable hospital, and comprehending instructions and risks related to hematoma, are indispensable aspects of safe discharge planning [22]. Thoughtful preparation is essential to ensuring the success and safety of such a program.

While our study provides valuable insight into post‐thyroidectomy neck hematomas, there are several limitations. The retrospective nature of this study inherently introduces limitations related to incomplete or missing data and the inability to control for confounding variables. Additionally, there was limited documentation regarding intraoperative blood loss or intraoperative hemostatic challenges. There were also no documented cases of clear inciting factors, such as increased thoracic pressure such as coughing or vomiting, which notably occurred in the postoperative period, but which was not documented. Finally, as with any rare outcome, the statistical power is restricted, particularly in subgroup analyses such as late‐onset hematomas. Our small sample size also precluded multivariate analysis, limiting the ability to adjust for potential confounders. A sample size estimation, assuming a significance level of 0.05 and a power of 0.8, indicates that approximately 196 cases would be needed to adequately power a study investigating the effect of postoperative elevated blood pressure on hematoma risk, and 134 cases would be required to study the impact of surgical extent on delayed hematoma risk. This exceeds the available cases in our data set and highlights the challenges of studying rare complications. Future studies would benefit from a larger cohort, with multicenter collaborations or extended study periods, to allow for more robust statistical findings.

## 5 Conclusion

Patients with comorbid type II diabetes, hypertension, and smokers should be carefully evaluated if consideration for outpatient thyroid is being contemplated. Further, increased vigilance should be emphasized in patients with Graves' disease. Additionally, patients with a pre‐induction systolic blood pressure > 160 mmHg, and patients who do not significantly respond to medication administration in the PACU should be carefully evaluated if consideration for outpatient thyroid is being contemplated. The same can be stated for patients undergoing extensive surgical procedures, such as total thyroidectomy and central neck dissection. By identifying critical patient factors necessary to safely execute thyroid surgery beyond the conventional in‐hospital overnight stay setting, this study seeks to optimize resource allocation and enhance patient outcomes within a public healthcare landscape.

## Ethics Statement

Ethics approval was obtained by the Medical‐Bioethics Research Ethics Committee (REC) of the Integrated Health and Social Services University Network for West‐Central Montreal (#2023‐3679).

## Consent

Patient consent was exempted by the REC.

## Supporting information


**Data S1.** Supporting Information.

## Data Availability

Emily Ajit‐Roger has full access to all the data in the study and takes responsibility for the integrity of the data and the accuracy of the data analysis. Additional data may be reasonably requested.
