# Supplementary material for: Factors Increasing the Likelihood of Postoperative Hematomas Following Thyroid Surgery
Source: Head Neck. 2025 Feb 12;47(7):1897–906. doi: 10.1002/hed.28096 (PMC12146824; doi:10.1002/hed.28096)
Supplement: Supplementary file 1 — Data S1. Supporting Information. [file HED-47-1897-s001.docx]

**Supplement 1. Surgeons**

| **Surgeons** | **Total thyroid surgeries** | **Thyroid hematomas** | **Incidence (%)** |
| --- | --- | --- | --- |
| Surgeon 1 | 25 | 0 | 0% |
| Surgeon 2 | 505 | 4 | 0.8% |
| Surgeon 3 | 522 | 9 | 1.7% |
| Surgeon 4 | 1193 | 7 | 0.6% |
| Surgeon 5 | 3257 | 10 | 0.3% |

**Supplement 2. Anesthesiologists**

| **Anesthesiologists** | **Total sample**  ***n* = 30 (%)** |
| --- | --- |
| Anesthesiologist 1  Anesthesiologist 2  Anesthesiologist 3  Anesthesiologist 4  Anesthesiologist 5  Anesthesiologist 6  Anesthesiologist 7  Anesthesiologist 8  Anesthesiologist 9  Anesthesiologist 10  Anesthesiologist 11  Anesthesiologist 12  Anesthesiologist 13  Anesthesiologist 14  Anesthesiologist 15 | 3 (10%)  1 (3.3%)  1 (3.3%)  1 (3.3%)  4 (13.3%)  1 (3.3%)  2 (6.7%)  1 (3.3%)  1 (3.3%)  1 (3.3%)  3 (10%)  7 (23.3%)  1 (3.3%)  2 (6.7%)  1 (3.3%) |
